# Peritoneal neuroendocrine carcinoma of unknown primary with markedly elevated alpha-fetoprotein: a case report

**DOI:** 10.3389/fendo.2026.1962119

**Published:** 2026-10-02

**Authors:** Jie Yang, Yaxin Zhang, Haifeng Que, Weibo Mao, Bitong He, Yinxia Wu

**Affiliations:** 1 Department of Infectious Diseases, The Fifth Affiliated Hospital of Wenzhou Medical University, Lishui Central Hospital, Lishui, Zhejiang, China; 2 Department of Gastrointestinal Surgery, The Fifth Affiliated Hospital of Wenzhou Medical University, Lishui Central Hospital, Lishui, Zhejiang, China; 3 Department of Pathology, The Fifth Affiliated Hospital of Wenzhou Medical University, Lishui Central Hospital, Lishui, Zhejiang, China; 4 Department of Medical Affairs, The Fifth Affiliated Hospital of Wenzhou Medical University, Lishui Central Hospital, Lishui, Zhejiang, China

**Keywords:** alpha-fetoprotein (AFP), large cell neuroendocrine carcinoma, neuroendocrine neoplasm, peritoneal neuroendocrine carcinoma, prognostic biomarker

## Abstract

**Background:**

Peritoneal neuroendocrine carcinoma(NEC) is primarily metastatic, with primary cases being exceedingly rare. The relationship between alpha-fetoprotein(AFP) and neuroendocrine carcinoma remains unclear. In this report, we present a patient with peritoneal neuroendocrine carcinoma who exhibited significantly elevated AFP levels. Extensive evaluation revealed no obvious primary site outside the peritoneum; however, occult gastrointestinal origin could not be entirely excluded. The tumor was thus classified as NEC of unknown primary, with a presumed primary peritoneal origin. The findings from this case suggest that markedly elevated AFP may be associated with aggressive tumor behavior and could serve as a potential marker for monitoring disease activity in peritoneal NECs, a hypothesis that warrants further investigation.

**Case presentation:**

A 51-year-old man with massive ascites and elevated AFP levels was admitted to the hospital. Liver enhanced magnetic resonance imaging and gastrointestinal endoscopy did not reveal any space-occupying lesions, while abdominal puncture did not detect tumor cells. The patient’s whole-body PET-CT revealed extensive peritoneal thickening and increased FDG metabolism. Subsequent laparoscopic exploration revealed numerous nodules and masses of varying sizes with a firm texture in the greater omentum, small intestine surface, mesentery, and parietal peritoneum. Immunohistochemistry of the omentum demonstrated positive results for AE1/AE3, Syn, CgA, and AFP, leading to a final diagnosis of large cell NEC of unknown primary, with a presumed primary peritoneal origin. Due to the presence of multiple peritoneal nodules, radical surgery was not feasible, and EP combined with camrelizumab was initiated. AFP levels showed an overall progressive increase, peaking at 5315 ng/mL on May 25, with a slight subsequent decline to 4948.4 ng/mL on June 20. Unfortunately, the patient discontinued chemotherapy after two cycles due to severe nausea and vomiting; he subsequently succumbed to the disease.

**Conclusions:**

We propose the hypothesis that dynamic changes in AFP levels could reflect tumor burden and treatment response in a subset of AFP-producing NECs. However, this hypothesis requires validation in larger cohort studies. It is also important to distinguish its potential role as a monitoring biomarker from an established prognostic biomarker.

## Background

Neuroendocrine neoplasms (NENs) are a rare type of tumor that originate from peptidergic neurons and neuroendocrine cells. They are characterized by neuroendocrine differentiation and the expression of neuroendocrine markers (1). The incidence rate in the United States is reported to be 6.98/100000 (1), and in the United Kingdom, it is 8.84/100000 (2). In 2019 (3), the WHO classified NENs into two categories, well-differentiated neuroendocrine tumors (NETs) and poorly differentiated neuroendocrine cancers (NECs), based on the degree of pathological differentiation. NECs is a rare malignant tumor that originates from a rare site. There have been only scattered cases reported worldwide, and currently, there are no definitive diagnostic or treatment criteria. This article presents a case of peritoneal neuroendocrine carcinoma with markedly elevated AFP, with the primary lesion considered to originate from the peritoneum, aiming to explore the role of AFP in evaluating the diagnosis, treatment, and prognosis of neuroendocrine tumors.

## Case presentation

A 51-year-old male patient with no significant past medical history was admitted to Lishui Municipal Central Hospital on March 9, 2023, due to persistent abdominal distension for one month, which had worsened over the past five days. The physical examination revealed abdominal protrusion with an abdominal circumference of 119 cm and shifting dullness was positive, while no abnormalities were observed in other physical examinations. On March 8, 2023, the patient’s AFP level was 991.70 ng/mL, and abdominal ultrasound revealed a significant amount of ascites. Subsequent blood tests conducted after admission showed a gradual increase in AFP levels (**Figure 1**). The patient’s liver function, renal function, routine blood test results, erythrocyte sedimentation rate, coagulation function, hepatitis B, HBV DNA, hepatitis A, hepatitis C, hepatitis E, HIV, syphilis, and routine stool test results were all within normal ranges.

**Figure 1:**
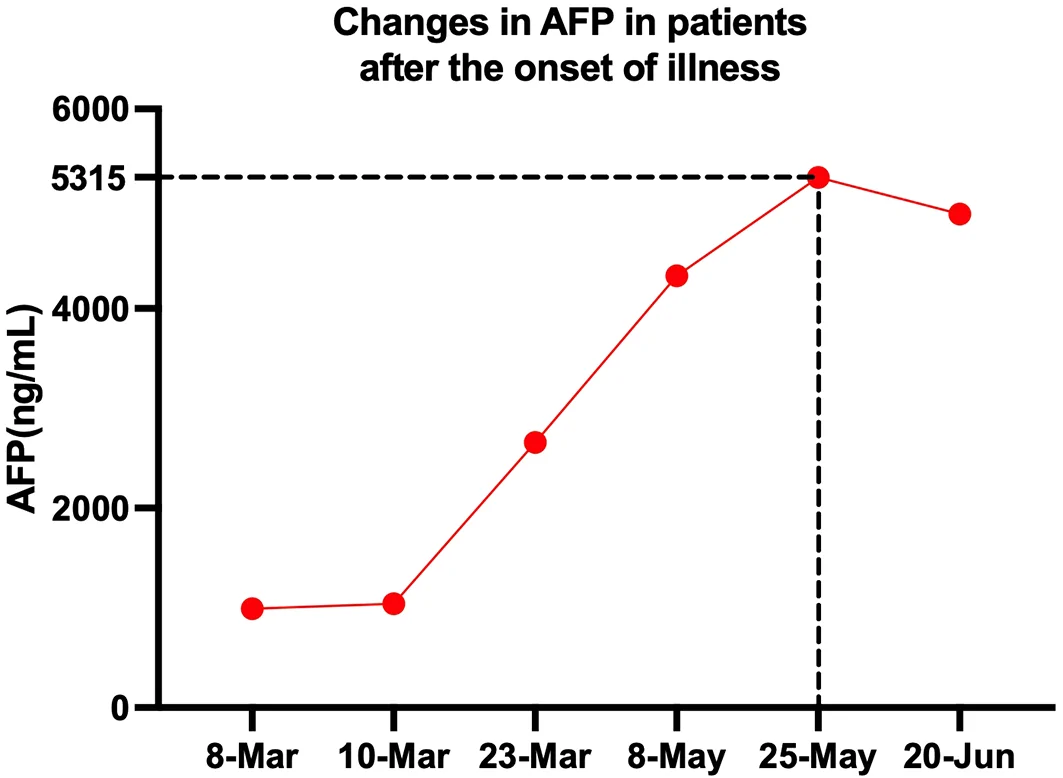
The AFP level showed an overall progressive rise from disease onset, with a slight decline at the last measurement.

Enhanced MRI of the patient’s liver revealed a slightly enlarged spleen with a significant amount of ascites and extensive thickening of the peritoneum. To determine the nature of the ascites, two abdominal puncture surgeries were performed. The AFP level in the ascites fluid was greater than 2000.0 ng/mL, but no tumor cells were detected in the shed cells of the ascites fluid. Pathological analysis of the ascites fluid revealed scattered atypical cells, mesothelial cells, and lymphocytes. Routine, biochemical, and culture tests of the ascites did not reveal any abnormalities. As a result, on March 14, 2023, the patient underwent a whole-body PET-CT scan, which indicated local thickening of the gastric antrum wall with slightly increased FDG metabolism. Further gastroscopic examination was recommended. The peritoneum showed extensive nodular thickening with increased ¹^8^F-FDG uptake (SUV_max_ = 12.4) (**Figure 2A**). There was mild enlargement of the lymph nodes in the right anterior diaphragm and diaphragmatic angle area, along with the accumulation of abdominal and pelvic fluid. Painless gastrointestinal endoscopy was performed on March 15, 2023, revealing chronic nonatrophic gastritis with erosion and a colonic adenoma. Capsule endoscopy and double-balloon enteroscopy were not performed because of patient refusal; this represents a limitation when attempting to rule out an occult primary small-bowel lesion.

**Figure 2:**
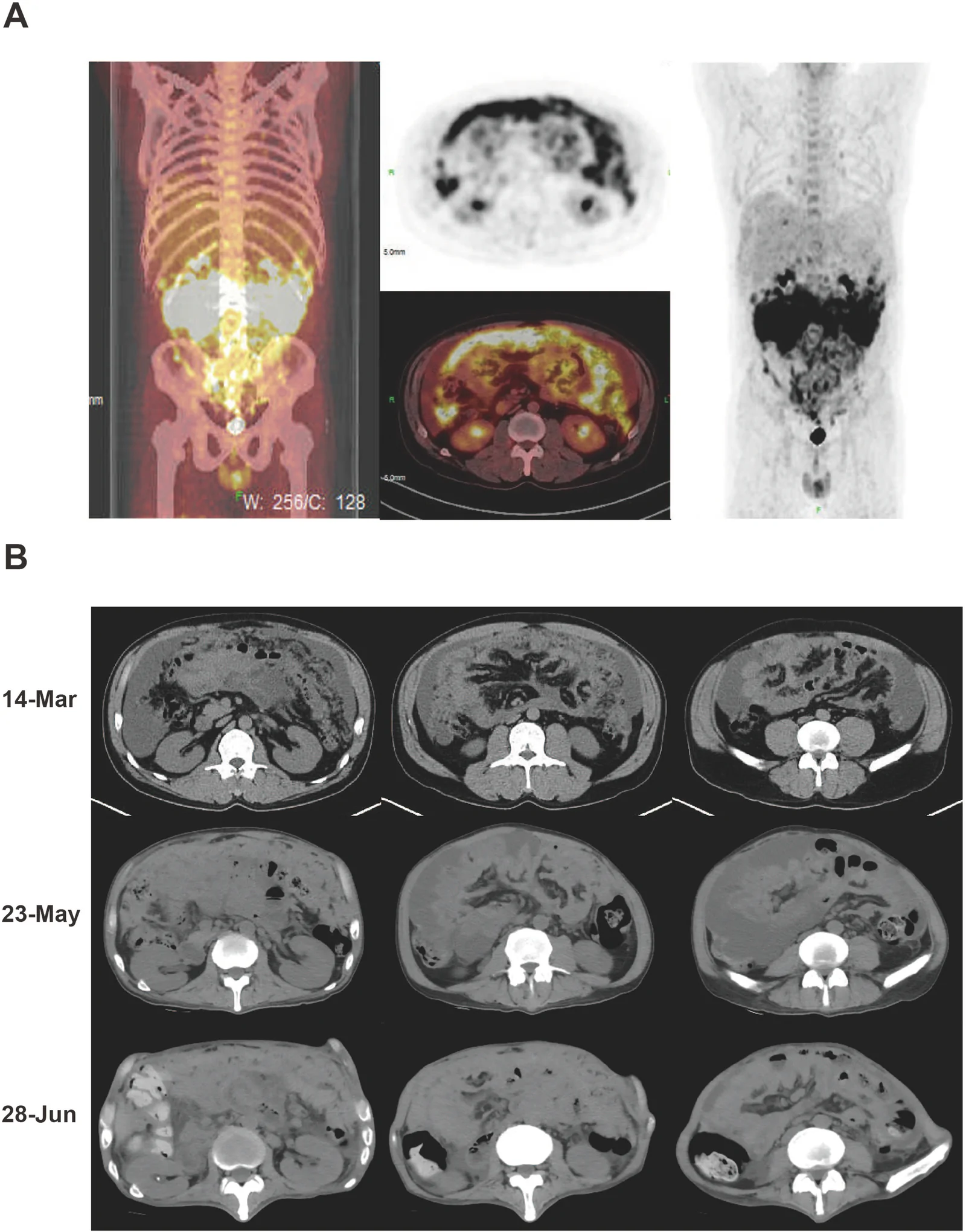
**(A)** The patient’s PTE-CT showed extensive nodular thickening of the peritoneum with increased uptake, and the maximum SUV_max_ was 12.4. **(B)** Serial abdominal CT scans obtained in March, May, and June. The images demonstrate extensive peritoneal thickening with haziness and multiple retroperitoneal lymphadenopathies, with progressive worsening in the extent and severity of peritoneal thickening over time.

After multidisciplinary team discussions, laparoscopic surgery was performed to explore the patient’s condition. The operation revealed a significant amount of yellow turbid ascites, approximately 6000 ml, in the abdominal cavity. Multiple nodules and masses of various sizes were observed in the omentum, surface of the small intestine, mesentery, parietal peritoneum, and other areas. Some masses were adherent with a firm texture (**Figure 3**). Pathology of the greater omentum revealed heterotypic cell nests in fibrous tissue, some of which resembled glandular structures. Focal degeneration and necrosis were also observed. Differential cancer infiltration was suspected (**Figure 4A**), Based on HE morphology and immunohistochemical results, a diagnosis of large cell neuroendocrine carcinoma was favored. Immunohistochemistry showed: Ki-67+ (80%), AE1/AE3 (+), Syn (+), CgA lesion (+), AFP partial (+), CD56 (-), CK7 (+), CK19 partial (+), CK20 (-), CDX2 (-), MUC2 (-), MUC5AC scattered (+), MUC6 (-), WT1 (-), and CR (-). *In situ* hybridization result: EBER (-). Additional immunohistochemical markers: BRG1 (+), INI-1 (+), S-100 (-), SATB2 (+), INSM1 (-) (**Figures 4B–F**). On April 5, 2023, FFPE-based capture next-generation sequencing (NGS), revealing tumor somatic mutations in TSC2 c.2072G>A (p.R691H) and RB1 c.748C>T (p.P250S). programmed death-ligand 1 (PD-L1) combined positive score (CPS) = 1. No potential germline genetic variations, genes related to positive response to immunotherapy, genes related to negative response to immunotherapy, genes related to hyperprogression of immunotherapy, or homologous recombination repair gene variations were detected. The final diagnosis was peritoneal large cell neuroendocrine carcinoma.

**Figure 3:**
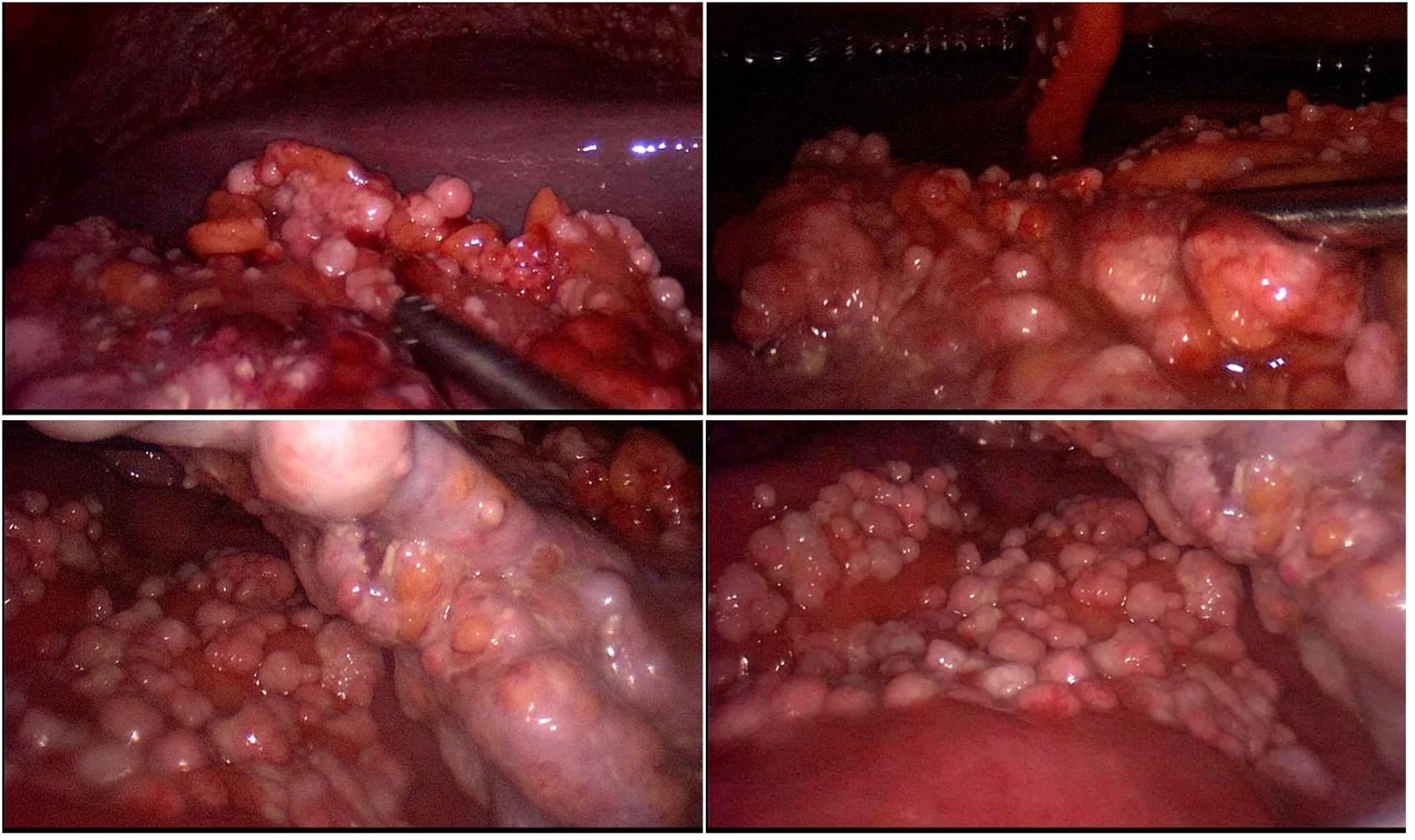
The operation revealed a significant amount of yellow turbid ascites in the abdominal cavity. Multiple nodules and masses of various sizes were observed in the omentum, surface of the small intestine, mesentery, parietal peritoneum, and other areas.

**Figure 4:**
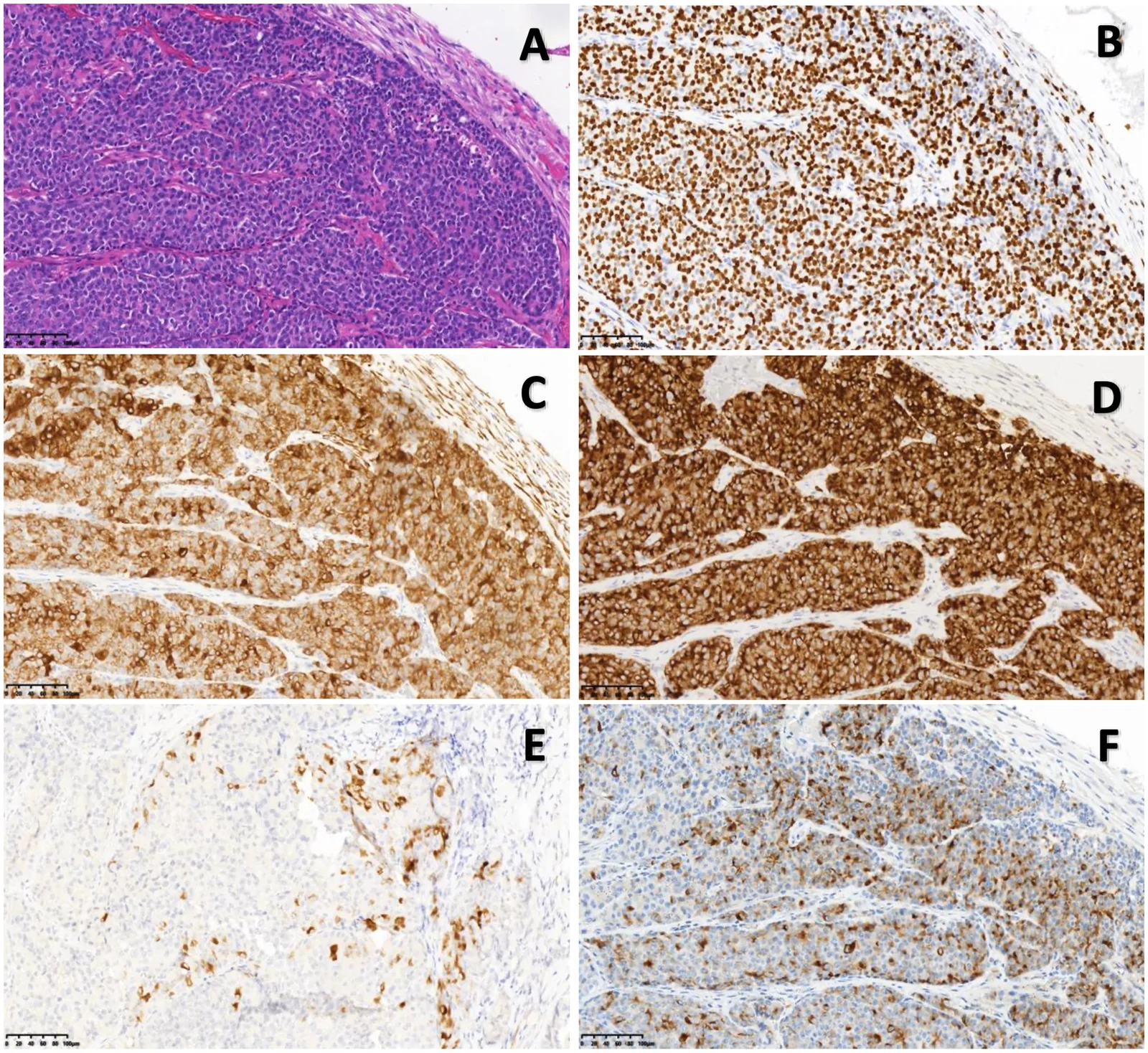
**(A)** H&E staining, ×200: Heterotypic cell nests can be seen in fibrous tissue, with some showing glandular-like structures and focal degeneration and necrosis. Immunohistochemistry: **(B)** Ki-67 immunostaining ×200; **(C)** AE1/AE3 immunostaining ×200; **(D)** Syn immunostaining ×200; **(E)** CgA immunostaining ×200; **(F)** AFP immunostaining, ×200.

Due to the presence of multiple masses in the patient’s peritoneum, surgical resection was not feasible. Based on the patient’s pathological findings and NGS results, the treatment regimens were as follows: Cycle 1 (April 6, 2023): cisplatin 120 mg IV day 1, etoposide 80 mg IV day 1 and camrelizumab 200 mg IV day 1; Cycle 2 (May 12, 2023): cisplatin 50 mg IV day 1-2, etoposide 140 mg IV day 1–3 and camrelizumab 200 mg IV day 1. However, despite two cycles of chemotherapy, the patient’s AFP level did not decrease and instead demonstrated an overall upward trend (**Figure 1**). During chemotherapy, the patient experienced severe gastrointestinal adverse effects, including intractable nausea and vomiting, making treatment intolerable. Accordingly, the patient declined further chemotherapy and chose home-based traditional Chinese medicine therapy. Follow-up abdominal CT scans performed in May and June both showed peritoneal thickening with haziness and multiple retroperitoneal lymphadenopathies. By June, peritoneal thickening had become more extensive, and the patient developed cachexia (**Figure 2B**). The patient ultimately died at the end of July. From disease onset, the AFP level exhibited a progressive rise, paralleling the increasing extent and severity of peritoneal thickening.

## Discussion

NENs are predominantly located in the gastrointestinal tract (60%) and lung (27%) (4). However, they can also occur in other areas, such as the central nervous system, pharynx, thyroid, skin, breast, and urogenital system (5). Most peritoneal and retroperitoneal neuroendocrine tumors are metastatic in origin (6). It is worth noting that NENs originating from the peritoneum are extremely rare, and only sporadic cases have been reported. Unfortunately, there is currently a lack of epidemiological data, as well as precise diagnostic and treatment standards for these patients.

Barnardo et al. reported the first case of a primary mesenteric NENs in 1984. The patient, a 74-year-old male, experienced intestinal obstruction caused by mesenteric carcinoid carcinoma. Four years later, an autopsy confirmed that the neuroendocrine tumor originated from the mesentery (7). Since then, more than 10 cases of NENs have been reported. Most of these tumors originate from the mesentery (8–17), with one originating from the greater omentum (18) and one from the lesser omentum (19). NENs are classified into low-grade inert tumors and high-grade invasive cancers based on their clinical behavior and histological manifestations (20). The histopathological differentiation of the above cases was well-differentiated, and the tumors were classified as NET G2 or lower. Two patients did not undergo surgery due to underlying systemic diseases and deteriorating condition, and eventually died (16, 17). The remaining patients underwent surgical resection, and their follow-up prognosis was good.

In 2019 (3), the WHO revised the gastroenteropancreatic neuroendocrine neoplasms (GEP-NENs) classification to address the limitations of the 2010 version (20). The revision introduced the “NET G3” diagnostic category, which specifically targets rare neuroendocrine epithelial tumors. NET G3 tumors are characterized by a Ki-67 proliferation index falling within the G3 range, which means a mitotic count of >20 per 10 high-power fields (HPFs) and/or a Ki-67 proliferation index exceeding 20%. These tumors are highly invasive and often lead to widespread metastasis and poor prognosis (21). Although both NET G3 and NEC show Ki-67 >20%, NET G3 retains neuroendocrine differentiation morphologically, whereas NEC is marked by severe structural disruption, extensive necrosis, conspicuous atypia, and a substantially worse prognosis. The evaluation of disease prognosis in GEP-NENs relies heavily on Ki-67 immunohistochemistry (22). A study by Milione et al. revealed that patients with well-differentiated tumors (NETs, n=24, Ki-67 <55%) and poorly differentiated cancers (NECs, n=30, Ki-67 <55%) had a significantly shorter median OS (43.6 months vs. 24.5 months). However, poorly differentiated cancers (NECs, n=82, Ki-67 >55%) had a significant decrease in median overall survival of only 5.3 months (23). In the case discussed here, the histopathological findings indicate poorly differentiated carcinoma, with Ki-67 accounting for approximately 80%. According to the 2019 WHO classification criteria, this tumor is classified as an NEC large cell type (LCNEC). In this case, the Ki-67 index was approximately 80%, and the patient’s overall survival was 5.5 months, which aligns with the aforementioned research findings.

In this case, multiple lesions were observed in various parts of the peritoneum, such as the omentum, surface of the small intestine, mesentery, and parietal peritoneum. PET-CT revealed significant thickening of the peritoneum and increased FDG metabolism; however, no abnormal FDG uptake was observed in other organs. Further examination through surgical exploration and gastrointestinal endoscopy did not identify any additional primary lesions. Immunohistochemistry analysis of the omentum biopsy showed positive results for Syn and CgA, indicating the presence of neuroendocrine tumors. Additionally, positive results for CK7 and CK19 suggested an epithelial origin. Histological examination of the tumor did not reveal any involvement of the lymph nodes, paraganglia, pancreas, or adrenal tissue. Notably, enteroscopy or capsule endoscopy was not performed; therefore, a small intestinal primary, particularly an occult jejunal or ileal lesion, cannot be entirely excluded. Given that metastatic peritoneal involvement by neuroendocrine carcinoma is far more common than a primary peritoneal origin, the evidence standard for definitively labeling this as “primary peritoneal” remains unmet. Consequently, we classify this entity more appropriately as peritoneal LCNEC of uncertain primary origin, with the peritoneum being the presumed primary site. Future similar cases should ideally include capsule endoscopy or double-balloon enteroscopy to exclude an occult small bowel primary.

Another characteristic of this case is the markedly elevated AFP level. AFP is a serum glycoprotein that is synthesized by the liver and yolk sac during fetal development. Its synthesis ceases after birth, and it is commonly used as a specific tumor marker in primary liver cancer. Recent studies have also shown that AFP levels increase in other malignant tumors, such as colorectal cancer, pancreatic cancer, and gallbladder cancer. Among these malignancies, gastric cancer is the most common (24). Compared with common tumors, AFP-producing gastrointestinal tumors are associated with a higher degree of malignancy, earlier metastasis, and poorer prognosis. AFP has been identified as an independent risk factor for prognosis that affects the prognosis of patients with AFP-producing gastric cancer (25–27), and it can also serve as a predictive factor for postoperative recurrence (28). In 2008, Shah et al. (29)established a database of neuroendocrine tumor cases and collected clinical data from 360 patients. Among them, 28 patients had >1.5-fold elevated AFP levels. Compared with the control group, tumors in patients with elevated AFP levels were more invasive, and elevated AFP was negatively correlated with survival. AFP-producing neuroendocrine tumors are associated with a high recurrence rate after treatment (30–32). Qiu et al. (31)reported a case of ovarian neuroendocrine cancer in which AFP levels decreased to within the normal range after surgery but increased again 6 months later. PET-CT scans revealed multiple metastases of the tumor, ultimately leading to death.

Previously reported cases of primary peritoneal neuroendocrine tumors (including mesentery, greater omentum, and lesser omentum) have not been associated with elevated AFP levels. This patient initially presented with abdominal distension and elevated AFP levels. Throughout the diagnosis and treatment process, AFP levels demonstrated a progressive increase. Despite undergoing two rounds of chemotherapy, AFP levels continued to rise, ultimately leading to the patient’s death within a short time frame. If the primary tumor in this case is confirmed to have originated from the peritoneum, this represents the first documented case of primary peritoneal neuroendocrine carcinoma associated with significantly elevated AFP levels. The mechanism underlying AFP elevation in non-hepatic malignancies remains unclear. Some studies have proposed that liver-specific genes are activated during the development of certain tumors, leading to liver-like differentiation of tumor cells and subsequent production of AFP (33–35). Additionally, there is evidence suggesting that tumor cells themselves may produce AFP, and AFP levels can rapidly decrease with treatment (36).

Our observation aligns with recent comprehensive evidence syntheses in the field. A recent narrative review by La Salvia et al. (37) systematically evaluated ten analytical studies comprising 1,222 GEP-NEN patients with available AFP measurements and concluded that AFP elevation, although uncommon, is consistently associated with biologically aggressive subsets characterized by high tumor grade, increased proliferative activity, and advanced disease stage. Importantly, that review emphasized that while elevated AFP correlates with poorer survival on univariate analysis, its prognostic signal diminishes or disappears after adjustment for established clinicopathological variables such as tumor grade and Ki-67 index. Consequently, AFP should not be regarded as an independent prognostic biomarker but rather as a contextual, adjunctive marker of aggressive tumor biology that requires integration within a multidimensional clinical and pathological assessment framework. In the present case, the markedly elevated AFP (peaking at 5,315 ng/mL) in the setting of a poorly differentiated LCNEC with a Ki-67 index of approximately 80% and extensive peritoneal dissemination fits well within the aggressive phenotype described in the literature. The progressive rise in AFP over the disease course paralleled the clinical trajectory of widespread, treatment-refractory disease. However, consistent with the current evidence base, we caution against interpreting this single observation as proof of AFP’s independent prognostic value. Instead, the dynamic AFP changes observed here are best conceptualized as a potential indicator of disease activity and tumor burden in this particular patient. It is also noteworthy that the patient never achieved documented remission; therefore, the concept of AFP as a predictor of recurrence is not applicable to this clinical course.

To date, there is no standardized diagnostic or therapeutic strategy for peritoneal neuroendocrine carcinoma (NEC). Radical surgery remains the first-line option for localized peritoneal NEC; however, this tumor is highly aggressive, rendering surgical intervention technically challenging and high-risk. For NEC of unknown primary, platinum-etoposide (EP) chemotherapy is the standard first-line regimen; nevertheless, it is associated with short-lived responses and frequent early progression, and durable survival benefit is rarely achieved with chemotherapy alone (38). The multicenter phase II NICE-NEC trial enrolled treatment-naive patients with advanced grade 3 neuroendocrine neoplasms, including cases of unknown primary origin; patients received nivolumab combined with carboplatin and etoposide, and 37.6% of participants achieved long-term survival exceeding 2 years, indicating that chemoimmunotherapy may induce durable antitumor responses in selected patients with extrapulmonary high-grade NEC (39). Meanwhile, a prospective multicenter phase II study is evaluating camrelizumab combined with chemotherapy as first-line therapy for advanced gastroenteropancreatic NEC in Chinese patients, aiming to assess its efficacy and safety (40).

In this case, PD-L1 expression was low (CPS = 1), and its predictive value for immunotherapy remains limited. Given the limited therapeutic options for diffuse peritoneal NEC of unknown primary, chemoimmunotherapy was considered to potentially offer clinical benefit; after full informed consent, EP combined with camrelizumab was administered as an exploratory treatment. Unfortunately, the patient ultimately had a poor outcome. Therefore, larger, high-quality studies are required to validate the survival benefit of chemoimmunotherapy in patients with diffuse peritoneal NEC of unknown primary.

## Conclusions

In this report, we present a case of peritoneal LCNEC with markedly elevated AFP levels and widespread peritoneal involvement. The origin was presumed to be the peritoneum, although an occult small intestinal primary could not be definitively excluded. The poorly differentiated histology and high Ki-67 index were consistent with an aggressive clinical course. Our observation raises the hypothesis that AFP may act as a dynamic indicator of disease activity in a subset of AFP-producing neuroendocrine carcinomas. We propose the hypothesis that dynamic changes in AFP levels could reflect tumor burden and disease activity in a subset of AFP-producing NECs, serving as an adjunctive contextual marker of aggressive tumor biology. However, its role as an independent prognostic biomarker is not supported by current evidence, and this hypothesis requires validation in larger cohort studies.

## Data Availability

The original contributions presented in the study are included in the article/**Supplementary Material**. Further inquiries can be directed to the corresponding author.
