# Supplementary material for: Peritoneal neuroendocrine carcinoma of unknown primary with markedly elevated alpha-fetoprotein: a case report
Source: Front Endocrinol (Lausanne). 2026 Oct 2;17:1962119. doi: 10.3389/fendo.2026.1962119 (PMC13672027; doi:10.3389/fendo.2026.1962119)
Supplement: Supplementary file 1 [file Table1.docx]

# Supplementary S1. Summary of Next-Generation Sequencing Findings

| **Parameter** | **Result** |
| --- | --- |
| Panel | Solid tumor 160-gene panel + PD-L1 (clone 22C3) |
| Platform | Illumina Hiseq4000 (capture-based NGS) |
| Sample type | FFPE tissue (omental biopsy) |
| Date performed | April 5, 2023 |
| Somatic mutations detected | TSC2 c.2072G>A (p.R691H); RB1 c.748C>T (p.P250S) |
| Germline pathogenic variants | None detected |
| Microsatellite status | MSS (score 0.0385; reference threshold 0.4) |
| PD-L1 expression (CPS) | 1.0 (positive; threshold ≥1.0) |
| Immunotherapy-positive predictive genes | No alterations detected |
| Immunotherapy-negative predictive genes | No alterations detected |
| Hyperprogression-related genes | No alterations detected |
| Homologous recombination repair gene alterations | None detected |
| Other actionable oncogenes (wild-type) | EGFR, ALK, KRAS, NRAS, ROS1, MET, ERBB2, BRAF, RET, NTRK1, NTRK2, NTRK3, KIT, PDGFRA, FGFR1, FGFR2, FGFR3, TP53, PTEN, PIK3CA |
